# Dosimetric effects of jaw tracking in step‐and‐shoot intensity‐modulated radiation therapy

**DOI:** 10.1120/jacmp.v13i2.3707

**Published:** 2012-03-08

**Authors:** Sarah Joy, George Starkschall, Stephen Kry, Mohammed Salehpour, R. Allen White, Steven H. Lin, Peter Balter

**Affiliations:** ^1^ Department of Radiation Physics The University of Texas MD Anderson Cancer Center Houston Texas USA; ^2^ Department of Bioinformatics and Computational Biology The University of Texas MD Anderson Cancer Center Houston Texas USA; ^3^ Department of Radiation Oncology The University of Texas MD Anderson Cancer Center Houston Texas USA; ^4^ The University of Texas Graduate School of Biomedical Sciences at Houston, The University of Texas MD Anderson Cancer Center Houston Texas USA

**Keywords:** IMRT, treatment planning, jaw optimization, normal tissue dose reduction

## Abstract

The purpose of this work was to determine the dosimetric benefit to normal tissues by tracking the multi‐leaf collimator (MLC) apertures with the photon jaws in step‐and‐shoot intensity‐modulated radiation therapy (IMRT) on the Varian 2100 platform. Radiation treatment plans for ten thoracic, three pediatric, and three head and neck cancer patients were converted to plans with the jaws tracking each segment's MLC apertures, and compared to the original plans in a commercial radiation treatment planning system (TPS). The change in normal tissue dose was evaluated in the new plan by using the parameters V5, V10, and V20 (volumes receiving 5, 10 and 20 Gy, respectively) in the cumulative dose‐volume histogram for the following structures: total lung minus gross target volume, heart, esophagus, spinal cord, liver, parotids, and brainstem. To validate the accuracy of our beam model, MLC transmission was measured and compared to that predicted by the TPS. The greatest changes between the original and new plans occurred at lower dose levels. In all patients, the reduction in V20 was never more than 6.3% and was typically less than 1%; the maximum reduction in V5 was 16.7% and was typically less than 3%. The variation in normal tissue dose reduction was not predictable, and we found no clear parameters that indicated which patients would benefit most from jaw tracking. Our TPS model of MLC transmission agreed with measurements with absolute transmission differences of less than 0.1% and, thus, uncertainties in the model did not contribute significantly to the uncertainty in the dose determination. We conclude that the amount of dose reduction achieved by collimating the jaws around each MLC aperture in step‐and‐shoot IMRT is probably not clinically significant.

PACS numbers: 87.55.D‐ 87.55.de 87.55.dk

## I. INTRODUCTION

**Figure 1:**
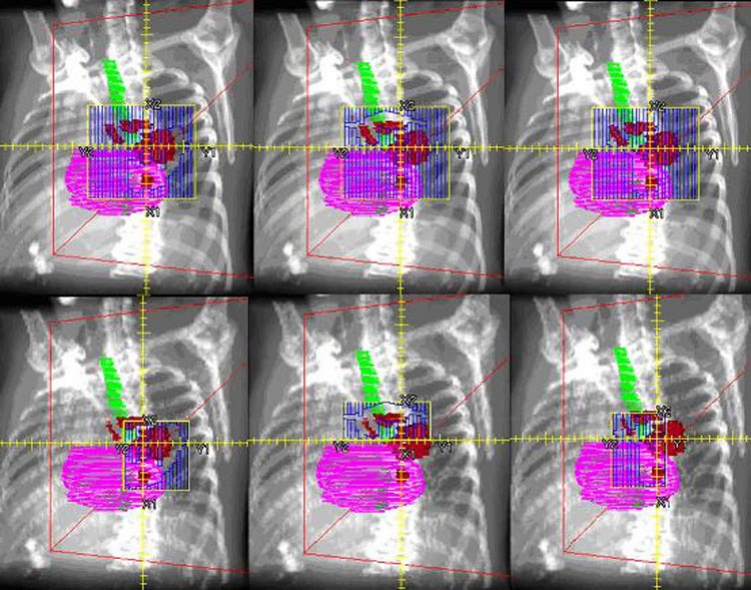
Three segments of an original step‐and‐shoot IMRT plan (top row) and the same three segments of a JTM step‐and shoot‐plan (bottom row). Target is in red, esophagus is in green, and the heart is in pink.

Intensity‐modulated radiation therapy (IMRT) can enable dose escalation by increasing conformity of the prescribed dose to the target while decreasing normal tissue doses in comparison to 3D conformal radiotherapy. However, IMRT may still deliver low doses to normal tissues outside of the radiation field due, in part, to leakage and transmission through the multi‐leaf collimators (MLC).^(^ ^1^ ^,^ ^2^ ^)^ On the Varian (Varian Associates, Palo Alto, CA) linear accelerators, the beam‐shaping collimation consists of upper and lower jaws followed by an MLC system, forming a tertiary collimation.^(^ ^3^ ^,^ ^4^ ^)^ This configuration allows the jaws to collimate to the MLC aperture in the directions parallel and perpendicular to the MLC. These Varian linear accelerators in particular, may have higher MLC transmission, as their MLCs do not attenuate radiation as much as the photon jaws.^(5)^ Although the jaws collimate to the MLC, this is only done based on the maximum extent of the MLC aperture for each beam; during the delivery of a given beam, the jaws do not move and do not tightly collimate each MLC opening. It might be possible to reduce these doses by moving the jaws to the edge of the MLC aperture for each segment of step‐and‐shoot IMRT, a technique we refer to as the jaw‐tracking method (JTM). The JTM is illustrated in Fig. 1, which shows three segments of a step‐and‐shoot IMRT beam with fixed jaw positions around the target, and then the same three segments with the jaws collimating each aperture, blocking transmission to the lung, esophagus, heart, and other normal tissues. In theory, JTM could be used to decrease normal tissue doses, or could be traded off for a higher prescribed dose which could potentially increase local control.

The purpose of this paper is to investigate if such a potential can be achieved with existent technology. In particular, we propose a method of implementing jaw collimation around each MLC aperture in step‐and‐shoot IMRT and test the concept in selective thoracic, pediatric, and head and neck patients.

## II. MATERIALS AND METHODS

Clinically approved step‐and‐shoot IMRT plans for ten thoracic, three head and neck, and three pediatric patients were used to implement the JTM. All patients in this study were enrolled in an institutional review board‐approved retrospective data collection protocol (2005‐0574). Thoracic patients were focused on due to the correlation between low doses in IMRT and thoracic toxicities; head and neck patients^(6)^ were evaluated because of the large number of segments typically found in their IMRT plans, and pediatric patients were evaluated because of the potential for integral dose sparing, which may reduce secondary malignancies.^(^ ^7^ ^–^ ^9^ ^)^ Patients who had relatively large treatment volumes (minimum planning treatment volume (PTV) was 218.0 cc, average jaw size $12\times 14\;{\text{cm}}^{2})$ were chosen as the ratio of the jaw area to the MLC area is larger and it was thought that more normal tissue would be spared with the JTM plan. All planning and measurements in this study were performed using 6 MV beams.

### A. Measurement of treatment planning system MLC transmission model accuracy

Measurements of MLC transmission were performed to validate the accuracy of the treatment planning system's $({\text{Pinnacle}}^{3}$, Philips Medical Systems, Madison, WI) transmission model. Calculations were first executed in the TPS and then measurements were carried out on a Varian linear accelerator (21EX) with a 120‐leaf MLC (Millennium 120, Varian Associates, Palo Alto, CA). Measurements were made with a $0.6\;{\text{cm}}^{3}$ ionization chamber (PTW N30001; Freiburg, Germany) placed at 1.5 cm depth with a source‐to‐surface distance (SSD) of 100 cm on central axis (CAX) in a polymethyl methacrylate phantom (PMMA). Six beams were delivered, three of them open fields of sizes $5\times 5\;{\text{cm}}^{2}$, $10\times 10\;{\text{cm}}^{2}$, and $12\times 12\;{\text{cm}}^{2}$, and the other three fields with the jaws at the same position but with the MLC covering the beam opening and the MLC leaf tips abutting under the jaw.

### B. Creation of JTM plans

The TPS was used to create the JTM plans. The commissioned clinical 6 MV photon beam model was modified to allow fractional monitor units (MU) and to include output factors down to $1\times 1\;{\text{cm}}^{2}$ so that the TPS could accurately calculate the JTM fields. The original clinically approved plan was copied and converted to the JTM plan with the aid of Pinnacle version 9 and MATLAB (The MathWorks, Inc., Natick, MA) scripts.

**Figure 2:**
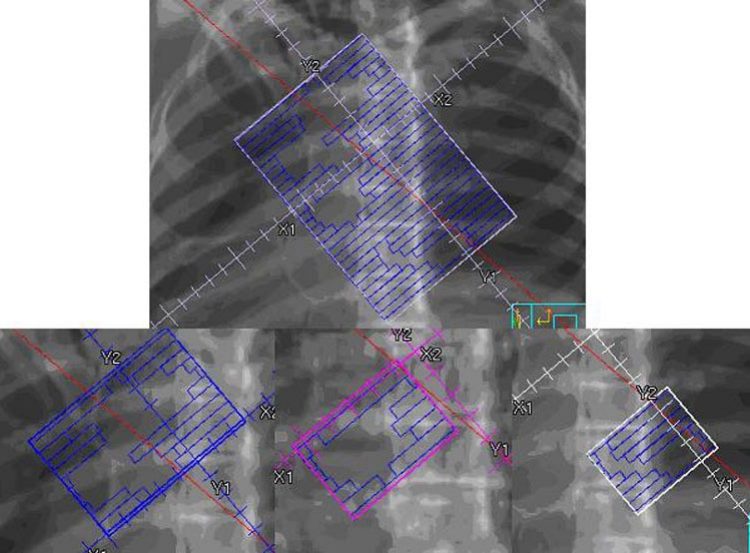
Original segment with three apertures (top) and the resulting three beams in the JTM plan each with one original MLC aperture (bottom).

Creating the JTM plan began with copying each original beam once for each segment in that beam. Segments were then deleted, so that each beam represented one of the original segments. Some segments contained more than one distinct aperture; these were further divided so that each beam in the JTM plan corresponded to one MLC aperture in the original plan. Figure 2 shows one segment with multiple apertures in the original clinical plan and the resulting three beams in the JTM plan, each with one aperture of the original segment.

Because jaws tolerances are 1 mm,^(10)^ they were pulled in to a 2 mm margin outside each MLC aperture to ensure the jaws remained outside each aperture. We determined the MUs for each beam in the JTM plan by taking the MUs for the corresponding segment (control point) from the original plan and rescaling it by the ratio of the collimator scatter factors $\left(S_{c}\right)$ for the original plan (denoted by the superscript *old*) to that of the JTM plan (denoted *new*) based on the original and JTM jaw sizes (Eq. (1)). This rescaling was done to maintain the original weighting per segment; because of uncertainties in the $S_{c}$, we found this was not adequate, and a full plan renormalization was then applied to maintain target coverage.

(1) $$MU_{ControlPo\mathrm{int}}^{new}=\frac{S_{C}^{old}}{S_{C}^{new}}*MU_{ControlPo\mathrm{int}}^{old}$$

**Figure 3:**
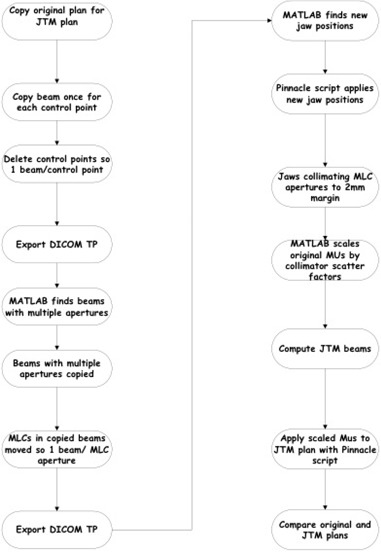
Flowchart documenting the process of creating a JTM plan.

Collapsed cone convolution was used as the dose algorithm for both JTM and original plans, and the prescribed dose in the JTM plans was changed from Gy/fraction to total MUs to match the assigned MUs. After rescaling the MUs with Eq. (1), we found that the target dose coverage was still insufficient, so the TPS prescribed dose was renormalized to achieve target dose coverage within 1% of the original plan. The renormalization was performed by increasing the total MUs until the target dose coverage was within 1% of the original plan; this ensured our weighting from Eq. (1) was retained. The target coverage was taken to be the patient's prescription achieved in the original clinical plan. For instance, if 70 Gy needs to cover 95% of the PTV and only 92% of the PTV was receiving 70 Gy after application of Eq. (1), we renormalized so that 70 Gy covered between 94% and 96% of the PTV. Figure 3 demonstrates the process of creating a JTM plan.

#### B.1 Evaluation of treatment plans

The percent volumes receiving 5, 10 and 20 Gy (V5, V10, V20, respectively) were determined for each patient, along with the average and maximum doses to appropriate head and neck structures in the head and neck patients; all information was obtained from cumulative dose‐volume histograms (DVHs). The values reported here are absolute differences between percentage volumes or calculated percentage differences between absolute doses.

### C. Off‐axis output factors

We postulated that the extra renormalization after application of Eq. (1) was necessary because our $S_{c}$ values were measured on central axis, while many of our JTM fields were off‐axis. To study this effect, we calculated off‐axis output factors in the TPS for field sizes $2\times 2\;{\text{cm}}^{2}$, $3\times 3\;{\text{cm}}^{2}$, $5\times 5\;{\text{cm}}^{2}$, and $8\times 8\;{\text{cm}}^{2}$ as a function of off‐axis distance. The off‐axis output factor was determined by calculating the dose to the center of the field, normalizing it by the dose to a 10×10 field on central axis, and dividing out the off‐axis factor.

## III. RESULTS

### A. Measurements

Absolute differences between MLC transmission measurements and calculations were found to be 0.2% or less (Table 1).

**Table 1:** MLC transmission measurements and TPS calculations.

*Field Size* $\left({\text{cm}}^{2}\right)$	*Measured Transmission (%)*	*TPS Calculated Transmission (%)*	*Absolute Difference (%)*
5×5	1.48	1.68	−0.20
10×10	1.53	1.60	−0.07
12×12	1.57	1.59	−0.02

### B. Treatment plans

The average number of beams in the JTM plans was 99.75 which ranged between 70 and 157 for all patients; the original average number of segments was 74.3 which ranged between 59 and 100. The JTM plans experience an average of 14.3% of jaw limitations, so about 14.3% of beams in each plan could not be fully tracked. After application of Eq. (1), the average renormalization of total MUs to maintain coverage was 1% to 3%.

**Figure 4:**
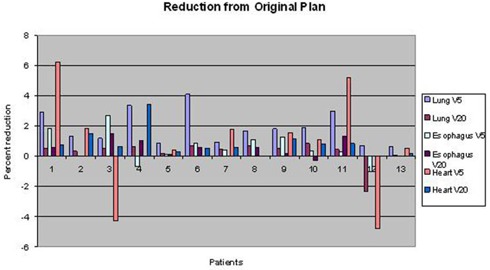
Absolute differences in relative V5 and V20 values between the original and JTM plan for thoracic and pediatric patients.

**Figure 5:**
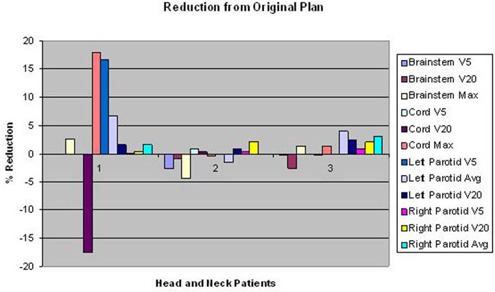
Absolute differences in relative volumes and percentage of difference in dose between the original and JTM plan for head and neck patients.

Figure 4 summarizes the dosimetric results for the thoracic and pediatric patients, showing the absolute differences in V5 and V20 values between the original and JTM plans for the lung, esophagus, and heart. The greatest lung V5 improvement due to application of the JTM was 4.1%, and the greatest lung V20 improvement was 0.8%. Figure 5 demonstrates the absolute difference in the percentage of volume and percentage difference in dose between the original and the JTM plan for head and neck patients.

**Figure 6:**
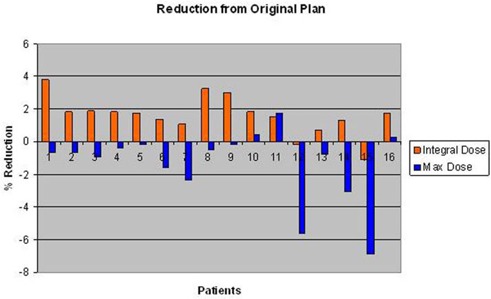
Percentage reduction in integral dose (orange) and maximum dose (blue) for all patients when the JTM plan was applied, versus the original plan.

The most improvement and detriment with the application of the JTM plan were for head and neck patient 1, with 18.0% improvement (decrease) in the maximum cord dose and a−17.6% detriment (increase) in the cord V20 (Fig. 5). Generally, the JTM plan reduced dose to normal tissues by about 2% (Figs. 4 and 5). Integral dose was also evaluated, and the amount of reduction achieved with the JTM plan is shown in Fig. 6. This figure also shows differences in the maximum dose to the target between the original clinical plan and the JTM plan. The integral dose was generally reduced by less than 2%, and few reductions in maximum dose were seen with the JTM plan (the maximum dose typically increased).

### C. Off‐axis output factors

**Figure 7:**
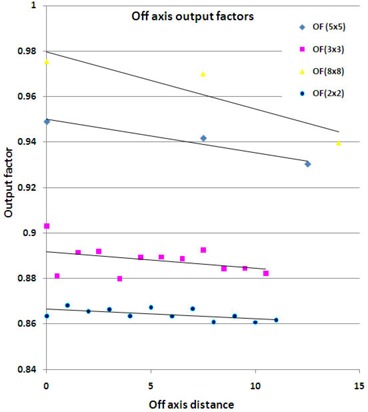
Output factors calculated in the TPS for field sizes of $2\times 2\;{\text{cm}}^{2}$ (blue), $3\times 3\;{\text{cm}}^{2}$ (pink), $5\times 5\;{\text{cm}}^{2}$ (purple), and $8\times 8\;{\text{cm}}^{2}$ (green). Dose to each field was normalized to a $10\times 10\;{\text{cm}}^{2}$ field on central axis, and then the off‐axis factor was divided out to obtain the output factors.

The results of the off‐axis output factor calculations (Fig. 7) demonstrate a 1% to 3% decrease in output with shifts away from central axis.

## IV. DISCUSSION

We believe our calculated dose reductions are accurate, despite the limitations of the TPS to calculate low doses. Our measurements of MLC transmission showed that our 6 MV model calculated a higher transmission value, but the 0.2% absolute difference is not substantial. The Pinnacle model for MLC transmission has a fixed value regardless of the MLC area exposed to the primary beam, whereas the output increases for increasing field sizes; we believe this explains the effective increase in calculated transmission with decreasing field size. Our measurements showed an apparent increase in MLC transmission with increasing jaw field size. This relationship is expected because, as more MLC area is exposed, more scatter in the MLC contributes to dose at our measurement point. The TPS does not model scatter within the MLC, so we did not see this effect in the calculations.

Our results demonstrate an overall (albeit, mostly small) reduction in normal tissue dose for the JTM plans compared with the original plans. Most patients had less than a 2% improvement in V5, V10, and V20 for their normal tissues. Previous work done by Prasad^(11)^ has shown a maximum change in dose output of 4.8% at a depth of $d_{\text{max}}$ on CAX for a 6 MV beam when the MLC and jaw apertures were $6\times 6\;{\text{cm}}^{2}$ and the jaw was changed to a $25\times 25\;{\text{cm}}^{2}$. Chapek et al.^(12)^ evaluated the effects of optimizing the collimator angle and jaw tracking for three prostate patients and found V60 differences between 3% and 9.6%. They included the collimator changes in the IMRT optimization in Varian Eclipse (Varian Medical Systems, Palo Alto, CA) TPS, which results in different DVH values with each optimization, though the authors tried to reduce this uncertainty by using a constraint template. It is also not clear whether their jaw optimization was on a beam, segment, or aperture basis. Schmidhalter et al.^(13)^ evaluated dosimetric effects of jaw tracking on dynamic IMRT for a prostate and head and neck case also using the TPS Eclipse. The largest difference in the prostate DVH was a 2.5% improvement with about a 3% increase in MUs. The largest difference found in the head and neck DVH was a 7% improvement with a 2.8% increase in MUs. Comparison of jaw, MLC, and combined fields for various dosimetric parameters have also been explored.^(^ ^14^ ^,^ ^15^ ^)^ All these studies were performed using Varian linear accelerators.

Previous work done concerning jaw tracking has yielded results in line with ours, though none have evaluated the effect on an actual clinical scenario of changing only the jaws on an MLC aperture basis in step‐and‐shoot IMRT.

For all structures in all patients, V5 typically showed the largest improvement and V20 the least improvement. There was no uniform shift in the DVH curves, indicating that improvement across a single structure varied widely for V5, V10 and V20. Likewise, there was no uniform shift across all normal tissue structure DVH curves for a single patient, indicating a patient may experience more sparing for one structure than for another. Some patients had one particular normal tissue improve to a greater extent than others when the JTM plan was applied, but we found no obvious relationship between improvement and parameters such as normal tissue volume or target location. We also could not find any parameters that clearly identified which patients would benefit most from the JTM. Comparison of the segment orientations with regard to the normal tissue structures, combined with comparison of the isodose lines, yielded some explanations for the results, but this cannot be quantified. Integral dose generally decreased for each patient but not by a substantial margin or in a predictable manner.

Some normal tissue structures experienced a negative response with the JTM plan indicating that the 5 Gy, 10 Gy or 20 Gy line expanded and covered more normal tissue. This result was unexpected, as the JTM plans should have reduced MLC transmission and, thus, normal tissue dose. However, because of the simultaneous reduction in target dose, the JTM plans had to be renormalized to maintain proper target coverage. Upon further investigation, we found that the prescribed (total MUs) and maximum doses of the JTM plans were higher than those of the original plans. The increase in some normal tissue volumes receiving 5 Gy, 10 Gy or 20 Gy was a result of the renormalization; we increased the amount of open field radiation for each segment, which pushed some of the isodose lines to encompass more normal tissue volume. This is a result of applying JTM after the original plan's IMRT optimization, so these results may not show the full potential benefit of JTM.

A weakness of this study is that the jaw tracking was added to existing clinical plans rather than being used in the initial optimization. This was done to allow a direct comparison between the same plans with and without the JTM. To study the effects of JTM being included in the optimization would require a statistical analysis of a large number of plans, as the expected improvements from JTM would be on the same order as the normal variations in plan quality. These results suggest that implementing the JTM on existing machines has little benefit, especially because of the difficulty in delivering these plans (owing to the fact that each aperture would be a separate beam). The ability to have the photon jaws track the MLCs on a segment‐by‐segment basis is expected to be available in the next generation of Varian accelerators (TrueBeam; Varian Associates, Palo Alto, CA); enabling this feature may have benefits beyond those seen in this work, especially if the jaw tracking is included in the dose optimization.

We expected $S_{c}$ to decrease with off‐axis distance because a different part of the extended source was exposed, and our calculations of off‐axis output factors demonstrated that this happened. The decrease in output observed was 1% to 3%, and the average renormalization needed after applying Eq. (1) was 3%; this result offers a reasonable, but only partial, explanation for why further renormalization was necessary.

## V. CONCLUSIONS

In general patients experienced an overall small dose reduction when the JTM plans were applied, compared to the original clinical plans. Those improvements in the JTM plan DVHs and integral doses were generally below 2%. This may not be of sufficient clinical benefit to warrant the implementation of this technique into current clinical practice. However, this approach to JTM relied on renormalization after IMRT optimization was complete, resulting in suboptimal JTM plans (including the expansion of certain isodose lines). These limitations would not have occurred if the optimization had included the jaw tracking, suggesting potential benefits in the JTM beyond the results reported here.

## ACKNOWLEDGMENTS

The authors wish to thank Drs. Bum Choi and Ramesh Tailor for their contributions to this work.
